# Analysis of Wheat Grain Infection by *Fusarium* Mycotoxin-Producing Fungi Using an Electronic Nose, GC-MS, and qPCR

**DOI:** 10.3390/s24020326

**Published:** 2024-01-05

**Authors:** Piotr Borowik, Valentyna Dyshko, Miłosz Tkaczyk, Adam Okorski, Magdalena Polak-Śliwińska, Rafał Tarakowski, Marcin Stocki, Natalia Stocka, Tomasz Oszako

**Affiliations:** 1 Faculty of Physics, Warsaw University of Technology, ul. Koszykowa 75, 00-662 Warszawa, Poland; rafal.tarakowski@pw.edu.pl; 2 Ukrainian Research Institute of Forestry and Forest Melioration Named after G. M. Vysotsky, 61024 Kharkiv, Ukraine; valya_dishko@ukr.net; 3 Forest Protection Department, Forest Research Institute, ul. Braci Leśnej 3, 05-090 Sękocin Stary, Poland; m.tkaczyk@ibles.waw.pl (M.T.); t.oszako@ibles.waw.pl (T.O.); 4 Department of Entomology, Phytopathology and Molecular Diagnostics, Faculty of Agriculture and Forestry, University of Warmia and Mazury in Olsztyn, Pl. Łódzki 5, 10-727 Olsztyn, Poland; adam.okorski@uwm.edu.pl; 5 Department of Commodity Science and Food Analysis, Faculty of Food Science, University of Warmia and Mazury in Olsztyn, Heweliusza 6, 10-719 Olsztyn, Poland; 6 Institute of Forest Sciences, Faculty of Civil Engineering and Environmental Sciences, Białystok University of Technology, ul. Wiejska 45E, 15-351 Białystok, Poland; m.stocki@pb.edu.pl (M.S.); ngierasimiuk@wp.pl (N.S.)

**Keywords:** gas sensor, application of e-nose, *Fusarium*, pathogen detection, odor differentiation, DON, ZEA

## Abstract

*Fusarium graminearum* and *F. culmorum* are considered some of the most dangerous pathogens of plant diseases. They are also considerably dangerous to humans as they contaminate stored grain, causing a reduction in yield and deterioration in grain quality by producing mycotoxins. Detecting *Fusarium* fungi is possible using various diagnostic methods. In the manuscript, qPCR tests were used to determine the level of wheat grain spoilage by estimating the amount of DNA present. High-performance liquid chromatography was performed to determine the concentration of DON and ZEA mycotoxins produced by the fungi. GC-MS analysis was used to identify volatile organic components produced by two studied species of *Fusarium*. A custom-made, low-cost, electronic nose was used for measurements of three categories of samples, and Random Forests machine learning models were trained for classification between healthy and infected samples. A detection performance with recall in the range of 88–94%, precision in the range of 90–96%, and accuracy in the range of 85–93% was achieved for various models. Two methods of data collection during electronic nose measurements were tested and compared: sensor response to immersion in the odor and response to sensor temperature modulation. An improvement in the detection performance was achieved when the temperature modulation profile with short rectangular steps of heater voltage change was applied.

## 1. Introduction

*Fusarium* fungi pose a direct threat to human health, for example, by infecting the eyes or fingernails. Fungi belonging to this genus are important in agriculture as pathogens of economically important crops. Two *Fusarium* species, *F. graminearum* and *F. oxysporum*, are included [1] in the list of the 10 most important pathogens of plant diseases. The species *F. graminearum* and *F. culmorum* are considered to be the main causes of *Fusarium* head blight (FHB), a disease that affects the ears of cereals such as wheat, barley, and other small-grain cereals [2,3,4]. Among other problems, this disease causes yield reductions and a deterioration in yield quality by contaminating the grain with mycotoxins, which are secondary metabolites of *Fusarium* [5].

EU legislation does not set a limit for the level of infection by fungi, but it does define very precise permissible limits for the maximum content of individual mycotoxins in food and feed (Commission Regulation (EC) No. 1881/2006 of 19 December 2006 setting maximum levels for certain contaminants in foodstuffs). For unprocessed cereals, the permissible DON mycotoxin content is 1250 μg/kg, and in the case of cereals for direct grain consumption (e.g., flour), it is 750 μg/kg. For the UAE mycotoxin, these values are 100 μg/kg and 75 μg/kg, respectively.

Accumulated and stored grain in silos allows the growth of fungi, including those of the genus *Fusarium*. Therefore, the presence of the pathogens poses a threat and makes the infected grain unsuitable for food or feed. For this reason, tools are needed for the rapid detection of fungal spoilage in collected samples. The detection of *Fusarium* fungi is possible using various diagnostic methods. The classical cultivation method, as well as immunological and genetic tests based on PCR reaction or direct DNA sequencing, can be performed with great success [5,6].

The advantages of molecular detection of contamination of plant material by fungi capable of synthesizing mycotoxins are repeatability, sensitivity (amounts of pg and even fg of DNA), and high accuracy [7]. However, these are typically laboratory methods that require a high level of cleanliness, which, in most cases, require the delivery of potentially infected samples to the laboratory and are also expensive. Therefore, alternative methods are still being sought. The goal of multiple research projects is the development of measurement techniques that will enable the testing of grain contamination with fungi, e.g., in a silo, and additionally, that will obtain information about contamination quickly and will make the testing itself less expensive.

Another group of proposed non-invasive methods for the detection of fungi involves the use of secondary volatile metabolites released during the growth of these organisms. The gold standard method for analyzing the chemical composition of gases is gas chromatography coupled with mass spectrometry (GC-MS). This allows us to identify individual chemical components by their relative composition. More advanced measurements also allow us to obtain quantitative information on the concentration of the components.

Fungal infestation of wheat is an increasingly serious nutritional problem in many countries around the world. *Fusarium* species (*F. cerealis, F. graminearum, F. culmorum*, and *F. redolens*) are particularly harmful pathogens due to their toxic metabolic products [8]. The most important mycotoxins include trichothecenes (DON, NIV, and T2 toxins), eniatins, fumonisins, and zearalenone [9,10]. Although DON is the least toxic mycotoxin, it is the most frequently detected and economically important mycotoxin produced by *Fusarium* [11].

The volatile compounds released by the *Fusarium* fungi were analyzed [12] by SPME–GC/MS, and an electronic nose was used to successfully distinguish (with an accuracy of more than 80%) between infected and non-infected wheat grains. Grain contaminated with toxigenic fungi can be analyzed by detecting and quantifying the associated mycotoxins using complex extraction procedures and analytical techniques. The air above the surface of wheat grain samples infested with *Fusarium poae* was analyzed for volatile metabolites using SPME–GC-MS, taking into account their development over time after inoculation. The chemical composition information was analyzed with an electronic nose to assess its ability to discriminate between differently contaminated samples.

Another method of detecting fungal infestation based on volatile organic compounds (VOCs) emitted is using electronic noses. The concept of such a device [13,14,15] is to use a series of non-specific gas sensors with an overlapping gas detection range. The discrimination between samples is based on the collected signals by applying pattern recognition algorithms. Since the sensors react to different chemical components with a non-linear response characteristic, the results of the electronic nose cannot be easily related to the analysis of the chemical composition by GC-MS methods when several volatiles are present in the emitted gases in a complex composition [16].

The quality of grain includes its appearance, nutritional value, and safety. As people’s standard of living has improved, grain quality issues have received more attention. Modern techniques are based on physical properties, including acoustic, optical, thermal, electrical, and mechanical. In particular, high-throughput sensory analyzers, such as electronic noses, tongues, or eyes, have unique advantages such as speed, indestructibility, accuracy, and efficiency [17]. The identification of fungal species by electronic noses has been demonstrated by Mota et al. [18]. The odors of different fungus-infested cereal grains [12,19], oilseed rape [20,21], and rice samples [22,23] were reported.

Our article is an attempt to improve the custom-made, low-cost device further. The electronic nose device used in the reported experiment was described in previous papers, where the construction of the electric circuit [24] and sensor chamber [25] were proposed. The current experiment’s main novelty focuses on verifying the various patterns of the sensor temperature modulation profiles and their optimization, allowing for a reduction in the measurement time.

Another goal of the experiment was to extend the application area of our device. Originally, we were looking for applications in forestry and are now looking in agriculture. The observed negative changes in the quality of the population’s diet seem particularly important and a priority for EU agencies such as the Food Safety Authority (EFSA). Cereals are an essential part of the classical food pyramid. Therefore, our attention is focused on new, fast, and effective methods to confirm (or not) its good quality for food purposes.

## 2. Materials and Methods

### 2.1. Sample Preparation

For the experiment, healthy grain without visible damage or discoloration (FHB) was selected as a control sample and was tested for the presence of *F. culmorum* and *F. graminearum* by qPCR and high-performance liquid chromatography (HPLC) methods to exclude DON and ZEA contamination.

Wheat seeds artificially infected with the fungi *F. culmorum* and *F. graminearum* were used to carry out the measurements. The fungal cultures *F. culmorum* (strain 13/2017DPF) and *F. graminearum* (strain IBL278f), from the collection of the Department of Entomology, Phytopathology and Molecular Diagnostics, Faculty of Agriculture and Forestry, University of Warmia and Mazury in Olsztyn, were split onto fresh potato dextrose agar (PDA) medium and then incubated at room temperature until the mycelium evenly covered the entire plate.

The prepared cultures were then used to inoculate wheat using the following procedure.

- Ten grams of grain were weighed and placed in sterile plastic Petri dishes with a diameter of 9 cm.
- Using a cork-borer with a diameter of 5 mm, fragments of the substrate together with the mycelium were cut out from plates overgrown with *Fusarium*.
- Then, 5 such fragments were placed on the plates between the wheat grains.
- The pieces with a clean PDA substrate were prepared similarly for the control variant.
- A total of 3 dishes were prepared for each variant: control, inoculated with the *F. culmorum* fungus, and inoculated with *F. graminearum*.
- Then, 3 mL of sterile distilled water (SDW) was added to each dish and incubated for 3 days at room temperature (about 22 °C).
- Every day, 1 mL of SDW was added to each dish, which allowed us to maintain a constant level of humidity of samples during the experiment.
- Four days after the contamination, infection was clearly visible and covered a substantial part of the Petri dish. Then, the electronic nose measurements were started.
- After the first 4 days of measurement, all dishes (control and infected) were replaced with fresh ones to avoid the risk of odor absorption from contaminated samples.

**Figure 1:**
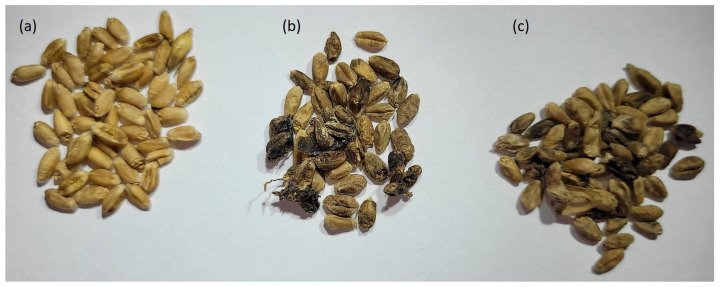
Photos of (**a**) healthy grain samples and grain samples infected by (**b**) *F. culmorum* and (**c**) *F. graminearum*. Samples were dried after measurements by the electronic nose.

Examples of grain infected by *Fusarium* fungi are presented in Figure 1.

After measuring samples with the electronic nose, the used samples were analyzed using qPCR and HPLC methods. Both infected and control samples were measured.

One comment is worth mentioning: The prepared samples of infected grain were similar when we could visually examine their coverage by fungi, as they all were infected at the same moment. However, the difference in mycelium amount could be noticed, and we could also observe that, during the experiment, they still grew, which introduced additional differences between them. For that reason, we could also expect that there will be various amounts of volatiles produced in each of the samples and each of the days of measurement. Such variability in the preparation of samples for an electronic nose measurement is desired, as the focus of the performed analysis is to find patterns of “odor fingerprints” and differentiate between healthy and infected samples regardless of odor strength. In further experiments, even more varied samples should be used, especially the samples with small amounts of fungi, which could be interesting for attempts to determine the level of infection or the threshold of detection.

### 2.2. Analysis of the Grain Infection Level by qPCR

#### 2.2.1. Isolation of DNA

- Infected wheat grains and non-infected grains as control (10 g of each trial variant in three biological replicates) were ground using 6775 Freezer/Mil (Spex Sample Prep, Metuchen, NJ, USA).
- Immediately after the wiping, the biological material was transferred to 1.5 mL Eppendorf tubes, and CTAB extraction buffer and RNase A (Promega) were added.
- The samples were incubated for 30 min in a ThermoMixer C with a speed of 350 rpm (Eppendorf, Hamburg, Germany).
- Every 10 min, samples were vortexed for 30 s using AMTAST Vortex 2 (Amtast Co. NORTH POINT HK).
- DNA isolation from infected wheat grain was carried out using the Maxwell 16 instrument and cartridges and reagents recommended by the manufacturer (Promega, Madison, WI, USA).
- The NanoDrop ND 2000C spectrophotometer (Thermo Scientific, Waltham, MA, USA) was used to determine the purity and quantity of the isolated DNA, which was then stored at 4 °C until further analysis.

#### 2.2.2. qPCR Quantification of *F. culmorum* and *F. graminearum* DNA in Wheat Grain

- The level of grain infection was determined using the qPCR method, which assessed the presence of DNA of both *F. culmorum* and *F. graminearum* fungi.
- The qPCR analysis was performed using the 7500 FAST system from Applied Biosystems in Waltham, MA, USA.
- The studies utilized specific primers for the EF1*α* gene that is present in both species. The primers used were FculC561 fwd: 5′-CACCGTCATTGGTATGTTGTCACT138-3′; FculC614 rev: 5′-CGGGAGCGTCTGATAGTCG-3′ for *F. culmorum* and FgramB379 fwd: 5′-CCATTCCCTGGGCGCT-3′; FgramB411 rev: 5′-CCTATTGACAGGTGGTTAGTGACTGG-3′ [26] for *F. graminearum* detection.
- The qPCR reaction mix consisted of 25 μL, containing 12.5 μL of the SYBR Green Universal PCR Master Mix (Applied Biosystems, Waltham, MA, USA), 10 pM of each primer, 4.5 μL of deionized water, and 5 μL of gDNA.
- The amplification process involved an initial denaturation at 95 °C for 5 min, followed by 40 cycles of denaturation at 95 °C for 15 s, primer annealing at 60 °C for 15 s, and strand synthesis at 72 °C for 1 min.
- The quantity of DNA isolated from each fungal species and genotype was calculated using calibration curves (made for each fungal species), which were determined in a previous study [5]. The Livak and Schmittgen [27] and Pfaffl [28] methods, with some modifications, were used to determine infection levels of grains.

### 2.3. Mycotoxin Analysis

#### 2.3.1. Standards and Chemical Reagents

Mycotoxin standards were purchased from Biopure^®^ Referenzsubstanzen GmbH, Tulln, Austria. Water for HPLC, organic solvents for HPLC, salts, and other chemicals were purchased from Sigma Aldrich (Saint Louis, MO, USA). The mobile phase HPLC water was purified using the Milli-Q system (Millipore, Bedford, MA, USA).

#### 2.3.2. Isolation of Mycotoxins from Cereal Grain

In this study, we used sample purification procedures to determine the levels of zearalenone (ZEA) and deoxynivalenol (DON) in the samples according to the methodology described by Okorski et al. [29]. The eluates were evaporated to dryness at 40 °C under a stream of nitrogen, and the dry residue was stored at 20 °C until HPLC analyses.

#### 2.3.3. HPLC Analysis

- High-performance liquid chromatography (HPLC) analyses were performed using a Nexera UHPLC instrument from Shimadzu (Kyoto, Japan), according to Visconti and Pascale [30].
- Chromatography of zearalenone was performed using a Synergy™ Hydro-RP column (4.6 mm × 250 mm, 4 μm) at a column temperature of 30 °C. The excitation and emission wavelengths were 274 and 440 nm, respectively. Acetonitrile–water–methanol (46:46:8, *v/v/v*) was used as the mobile phase with a 1 mL/min flow rate.
- Quantification of ZEA was performed by measuring the peak areas at the ZEA retention time according to the relevant calibration curve (correlation coefficient r = 0.9998). The limit of zearalenone detection was 0.003 mg/g, with a recovery range from 96 to 99%. The relative standard deviation (RSD) value was below 1%.
- Deoxynivalenol was quantified by high-performance liquid chromatography (HPLC) using a Nexera UHPLC instrument from Shimadzu (Kyoto, Japan).
- Analytical determination of deoxynivalenol was carried out after chromatographic separation using a Gemini^®^ C18 Phenomenex HPLC column (4.6 mm × 150 mm; 5 μm) at 30 °C. Detection was carried out using a detector set to a wavelength of 218 nm. DON was eluted from the column with a 10% water solution of acetonitrile (flow rate 0.6 mL/min) with retention times of 8.63. The limit of deoxynivalenol detection was 0.002 mg/g, with a recovery range from 95 to 97%.

### 2.4. Measurement of Volatiles Emitted by *F. culmorum* and *F. graminearum*

- Volatile organic compounds (VOCs) emitted by *F. culmorum* and *F. graminearum* were investigated by headspace solid-phase microextraction and gas chromatography coupled with mass spectrometry (HS-SPME/GC-MS).
- Petri dishes with *F. culmorum* and *F. graminearum* were sealed using parafilm and heated for 30 min at 40 °C. The parafilm was pierced by a needle, and the SPME fiber (Supelco, Bellefonte, PA, USA) with stationary phase DVB/CAR/PDMS (divinylbenzene/carboxen/polydimethylsiloxane) was exposed to the headspace gas phase. The time of fiber exposition was 30 min. Afterward, the fiber was placed for 10 min in an injector of the GC-MS device.
- The GC-MS measurements were performed on an Agilent 7890A gas chromatograph coupled with an Agilent 5975C mass spectrometer (Agilent Technologies Inc., Santa Clara, CA, USA). The injection port was worked in splitless mode at a temperature of 250 °C. The separation of compounds was performed on a capillary column HP-5MS (30 m, 0.25 mm, 0.25 μm), and the helium flow rate was 1 mL/min. The starting temperature of the column was 35 °C and increased at a rate of 5 °C/min to 250 °C. The temperatures of the ion source and quadrupole were 230 °C and 150 °C, respectively. The ionization energy in the mass spectrometer equaled 70 eV. Detection was performed for a range of 29–600 units in full scan mode.
- After integrating the peaks from the chromatogram, the percentage content of the volatile compounds in the total ion current (% TIC) was calculated. The mass spectra and retention indices were used for the identification of volatiles.
- Mass spectrometric analysis was performed using the NIST (2020) and Wiley (2020) mass spectral libraries, as well as the atlas by Adams (2007) and Tkachev (2008). The retention indices of the compounds were calculated considering the retention times of the alkanes.
- In an independent run, the sample of C5-C40 n-alkanes (1 μL) was injected into the GC column and separated using the conditions previously described for GC-MS analyses of volatile compounds. Experimental retention indices ($RI_{exp}$) were determined with use of the following expression:
  $$RI_{exp}=100\left[n+\left(t_{x}-t_{n}\right)/\left(t_{n+1}-t_{n}\right)\right],$$
  where *n* is the number of carbon atoms in the n-alkane, $t_{x}$ is the retention time of the compound, $t_{n}$ is the retention time of the alkane eluting immediately before the compound, $t_{n+1}$ is the retention time of the alkane eluting directly after the compound. The experimental retention indices ($RI_{exp}$) were compared with the literature retention indices ($RI_{lit}$) posted in the database mentioned above.

### 2.5. Electronic Nose Measurements

#### 2.5.1. Electronic Nose Device

**Figure 2:**
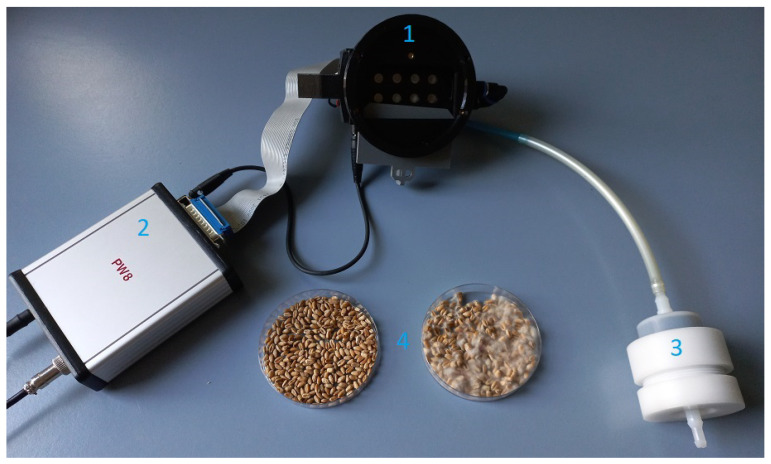
Measurement setup of the used electronic nose. (1) Sensor chamber, (2) control unit, (3) charcoal air filter, (4) measured samples of healthy (left) and infected (right) grains in Petri dishes.

In Figure 2, one can see a photo of the electronic nose device used, together with two samples of measured grain in Petri dishes. Details of the electrical circuit [24] of the electronic nose and the construction of the sensor chamber and operation principle [25,31] were described in previous papers.

- The electronic nose consists of two main parts. The first part is a set of Figaro Inc. (Osaka, Japan) [32] TGS series MOX sensors (Table A1 in Appendix A), which are placed inside the sensor chamber and consist of a probe that can be applied to the measured sample. The second part is the main electronic unit, which is connected to the computer.
- The gas sensor chamber is designed to expose the sensors to the measured volatiles by opening the shutter. When the sensor chamber is closed, a pneumatic system provides a clean air flow to clean the sensors between measurement cycles and allows measurement of the baseline levels of the sensor’s signals.
- Communication between the sensors and the computer is controlled by the ATmega 328P-PU microcontroller.
- The MOX sensors require high-temperature conditions of several hundred degrees Celsius for proper operation of their sensing element, which is achieved by internally installed heating elements supplied with 5 Volts of electrical power, according to the manufacturers [32]; sensors strongly depend on the conditions of the heating voltage [33]. Our electronic nose circuit is equipped with a stabilization of the heating voltage at a required level [24]. In addition, the heating voltage of the sensor can be modulated, which enables the modulation of the operating temperature.
- The electronic nose is equipped with a separate circuit that detects the sensor’s response signal, i.e., the change in its conductance after changing the measurement conditions. The sensor conductance is estimated by measuring the voltage across a connected resistor [34]. The transient sensor response is recorded, and the sensor measurement cycles are repeated every 0.75 s.

With the electronic nose used, two methods can be used to generate a transient sensor response: (1) opening the sensor chamber, which changes the conditions of the gas composition in which the sensors are immersed from pure air to the measured odor, and (2) changing the operating temperature by changing the sensor heater voltage. We refer to the first method as the adsorption phase, since such a physical effect occurs, and the second method as the temperature modulation phase.

#### 2.5.2. Collecting Electronic Nose Signals

**Figure 3:**
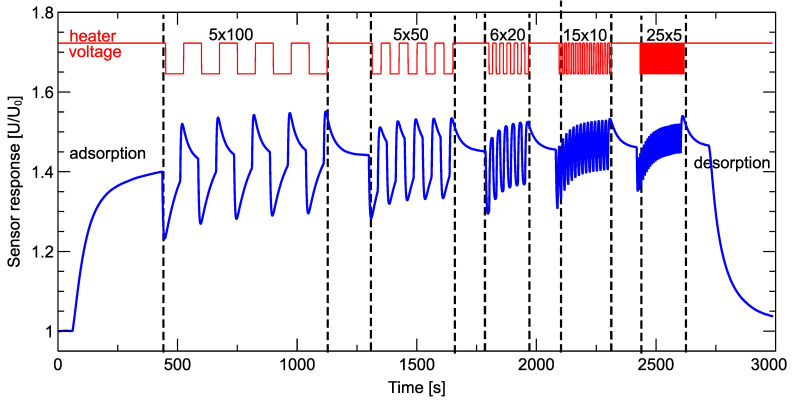
Example of a sensor response during one measurement cycle. The shape of the heater voltage modulation is plotted in red color above. Several series of sensor heater modulations with different frequencies are marked by delimited dashed vertical lines. The number of repetitions and duration of a modulation step (sensor reads units of 0.75 s) of each phase of the heater voltage modulation are indicated in the figure.

In Figure 3, we give an example of the shape of the signal collected by the electronic nose sensor during one measurement cycle.

The presented curve is the voltage measured at the resistor serially connected to the sensor [34] and represents the sensor’s conductivity. This quantity can be presented in arbitrary units, as the important quantity is the relative value $U/U_{0}$, where $U_{0}$ is the baseline level. What is important is that the baseline level has different meanings for two methods of creating the transient response. For the data collected during the adsorption phase, the baseline level is the voltage determined when the sensor was exposed to clean air. For the temperature modulation phase, the baseline level is the voltage measured just before the start of the modulation cycle.

The measurement procedure was carried out according to the following scheme, the parts of which can be distinguished in Figure 3:

- The sensor chamber with a closed sensor cover was placed on the open pan, and the software to control the device was started.
- The first 100 measurement units (75 s) were performed with a closed sensor chamber with a forced flow of clean air. This phase lasted 75 s and was used to determine the baseline. The collected response in this area was also needed to ensure that the response curve remained flat, meaning that the condition of the sensor was stable and the sensor was clean enough after the previous measurement cycle. (Clean air baseline collection phase.)
- After the first phase, the shutter of the sensor chamber was opened manually, and we recorded the reaction of the sensors to the presence of the measured gas. This phase lasted a total of 3546 measurement units and was divided into several phases of modulation of the heating voltage of the sensors. During this time, the sensors remained exposed to the measured gas sample.
  – The first data acquisition phase lasted 500 time units (6 min and 15 s). This duration was chosen so that the sensor would reach a quasi-steady state and its response would not change significantly. We determined this time based on several previous test measurements.
  – In the second phase, the sensor heater voltage modulation phase, we switched the heating voltage every 100 time units (75 s) and repeated the modulation periods 5 times. A rectangular modulation shape was used, with the upper nominal voltage recommended by the manufacturer at 5.0 V and the lower nominal voltage at 4.5 V.
  – After that modulation cycle, we included an interval of 150 time units (2 min 30 s) at a nominal voltage of 5.0 V, which allowed us to achieve steady-state response conditions of the sensors in the measured gas conditions.
  – We then performed the sensor heater voltage modulation cycles with steps of durations of 50, 20, 10, and 5 time units, which were repeated 5, 6, 15, and 26 times, respectively. The rectangular shape of these modulations with voltage levels of 5.0 and 4.5 V was used.
  – After each such cycle of several repetitions of modulation periods, we included intervals of 150 time units (2 min 30 s) at a nominal voltage of 5.0 V, allowing us to reach steady-state response conditions of the sensors.
- The last phase of the measurements consisted of closing the sensor chamber and pumping clean air into it to clean the sensors. The responses of the sensors were still recorded but not used for further analysis. This phase lasted 600 measurement units (7 min 30 s). (Desorption phase.)

**Figure 4:**
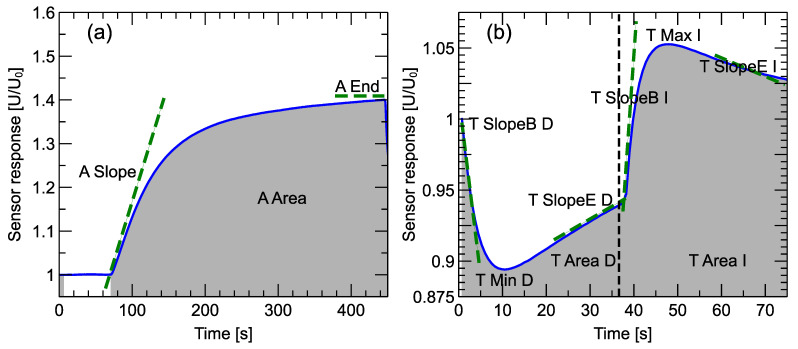
Schematic representation of features extracted from a sensor response curve. (**a**) Adsorption phase, and (**b**) sensor temperature modulation phase. The heater modulation profile is plotted in the subfigure. The magnitude of the response is represented as $U/U_{0}$, where $U_{0}$ is the baseline level measured at the beginning of the considered phase of the measurement. A description of the features is available in Table 1.

**Figure A1:**
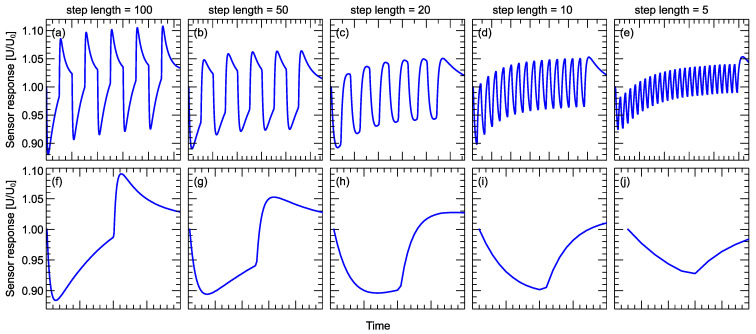
Example of a sensor response to heater voltage modulation of various frequencies. The top row of subfigures (**a**–**e**) presents all collected repetitions of modulation; the bottom row of subfigures (**f**–**j**) presents the first repetition of modulation. The step length indicated above the subfigures is represented in the sensor’s reads (0.75 s).

The measured sensor response to a small drop in the heater voltage causes the measured response voltage to also abruptly drop. After a short period, it reaches its minimal level and then starts to increase to teach a different stationary level (Figure 4 and Figure A1 in Appendix B). The sensor response to the increase in the heater voltage is analogous. Firstly, the read response abruptly increases, and after reaching the maximum level, it starts to decrease until it reaches some stationary level.

The rationale behind such an approach to the choice of the duration of modulation steps of the heater response was to verify the influence of that parameter on the ability to differentiate between healthy and infected samples using the electronic nose data. As we can notice from examining the example of a sensor response in Figure A1, the modulation frequencies were chosen in such a way that the switching to the following voltage level occurred before the response reached a stationary state. There is also another effect that could be observed. The first repetition of heater voltage modulation was different than the subsequent repetitions (Figure 3, Figure A1). As we could also observe in Figure A1 (Appendix B), such a difference is much more pronounced for a higher frequency of modulation (shorter steps), and more repetitions were needed to reach the stationary repetition when the subsequent one was very similar to the previous one.

#### 2.5.3. Extracting of Features Describing Sensor Response Curve

In Figure 4a, we present an example of a sensor response during the adsorption phase of the sensor response when the measured odor enters the sensor chamber. During that time, 500 sensor response values for each sensor were collected. Before using these data for further analysis of building machine learning models, we performed a preprocessing step of the extraction of significant features, which can describe the shape of the curve. For that part of the curve, we used three features: the slope of the response at the beginning of the observation period, the magnitude of the response reached at the end of the observation period, and the average response during the observation period.

In Figure 4b, we present an example of sensor response during one sensor heater voltage modulation period. The period includes both voltage decrease and voltage increase steps, and similar features were extracted from both. In these cases, the number of observations collected during the modulation period depended on the modulation step length and varied from 200 to 10, but 8 features for each sensor data were always extracted and used for further analysis. The list of features extracted from the response curves is presented in Table 1.

#### 2.5.4. Experiment of Measurements by the Electronic Nose

During the experiment, a complete series of measurements was carried out on all dishes daily. Before the measurements began, the order of the bowls was determined using a random generator.

In total, the measurements were carried out over six days. On each day of the experiment, the odors were collected only once from all Petri dishes (three dishes for each variant). The measurements were taken daily to detect possible changes related to the development of the fungus or changes in the moisture content of the seeds themselves. The first part of the measurements was carried out from 24 to 27 October 2023. After these measurements, all trays were replaced with fresh ones, and measurements were carried out for two more days (30 to 31 October 2023).

According to the manufacturer’s recommendation, the sensors were preheated at least one week before the measurements and were connected to the power supply throughout the experiment.

#### 2.5.5. Machine Learning Classification Models Using Electronic Nose Data

##### Random Forest Classification

Signals collected as an electronic nose response can be used for building machine learning classification models to differentiate between studied sample types. One of the most popular classification model algorithms is Random Forest (RF) [35], which was used in our studies.

The RF method is an ensemble model, which consists of creating many weak classifiers of decision tree models. Each individual is trained independently on a portion of the training data set. After that, the final model is constructed as the average of the results of these classification trees. The final ensemble model allows the modeling of nonlinear patterns in the data, usually achieves significantly better performance than the individual models, and is less prone to overfitting than many other models.

One of the interesting advantages of the RF technique is the ability to extract, during the training process, the so-called out-of-bag (OOB) score. The idea behind this is that, since the training, only subsets of the training data sets are used for the creation of individual decision trees, the remaining part of the data can be used for an independent estimation of the model performance. The OOB score can be calculated for various considered model performance measures, such as accuracy, precision, and recall. The OOB score concept is very similar to the cross-validation approach, and it has been demonstrated that OOB results converge to leave-one-out cross-validation [36].

##### Target and Predictors and of the Classification Models

The main goal of the electronic nose measurements in the studied case was an examination of the possibility of detection of wheat infection by the *Fusarium* fungi. We used two species of these pathogens to prepare the samples, but for the classification analysis, we trained binary classification models, allowing differentiation between healthy and infected samples.

As the predictors for the models, we used features extracted from the six sensor response curves, as described in Section 2.5.3. For the case of the sensor heater temperature modulation, we extracted these features from the first repetition of the modulation and the last one. The set of features used for the model training depended on the type of analysis that we performed.

There were several series of models that we trained, the performance of which we compared, which differed in the list of used predictors:

- Model using features extracted from the adsorption phase of the sensor response.
- Models using sensors’ responses from the first repetition of a given frequency’s heater temperature modulation phase.
- Models using sensors’ responses from the first and last repetition of a given frequency’s heater temperature modulation phase.
- Models using features extracted from the adsorption phase of the sensors’ responses and the heater temperature modulation phase of the responses.

##### Classification Model Performance Measures

Three of the most commonly used measures of the model’s performance were calculated in our studies.

- **Accuracy**, which reflects the proportion of correctly classified cases compared to the total number of cases.
- **Precision**, which reflects the ratio of the number of correctly classified cases compared to the number of cases classified as belonging to this category. That means that this measure is focused on the confidence that the classified record truly belongs to this category.
- **Recall**, which is the ratio between the number of cases correctly classified in proportion to the total number of cases in this category. It reflects the possibility of detecting observations belonging to that group and is not penalized when observations from the opposite category are incorrectly classified.
  $$\begin{array}{ccc} accuracy & = & \frac{tp+tn}{tp+tn+fp+fn},\\ precision & = & \frac{tp}{tp+fp},\\ recall & = & \frac{tp}{tp+fn}, \end{array}$$
  where the components of the confusion matrix (tp: true positive, tn: true negative, fp: false positive, fn: false negative) reflect whether the observations are classified correctly or not by the model.

In our implementation, the model training procedure was repeated 100 times with various random number generator seeds to obtain more reliable outcomes, and the final results of the model performance measures were averaged.

##### Software Packages for Data Analysis

The data analysis presented in this manuscript was performed using computer programs prepared in the Python 3.10 language. The scikit-learn package [37] was used for machine learning modeling and model performance estimations.

## 3. Results of the Experiments

### 3.1. qPCR and Mycotoxins Analysis

**Figure 5:**
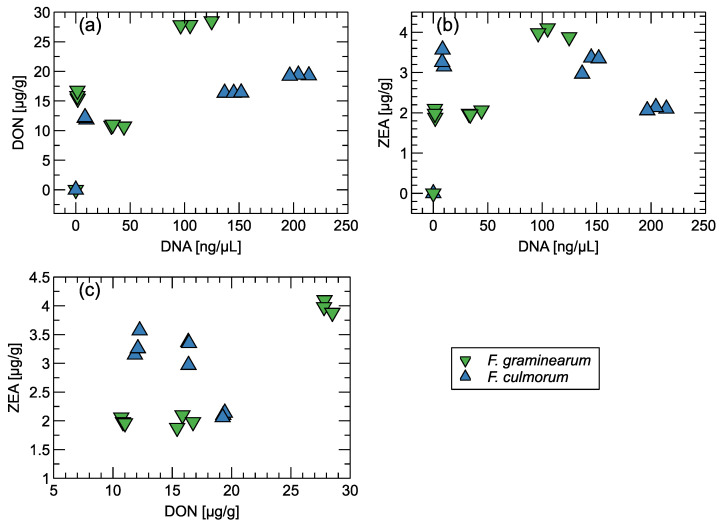
Distribution of the concentration of toxins DON, ZEA, and fungi DNA in the measured samples. (**a**) DON vs. DNA, (**b**) ZEA vs. DNA, (**c**) ZEA vs. DON.

In Figure 5, we present the results of the DON and ZEA toxin level measurements and the quantity of DNA detected in the samples used in the experiment.

Grain infection was clearly visible (Figure 1). During the analyses performed with the electronic nose, the grains were successively overgrown with mycelium. After a week of that part of the experiment, the grain damaged by the infection was dried, and then the presence of fungal DNA was analyzed and the concentration of DON and ZEA mycotoxins was determined. As a result of qPCR analyses, a high level of fungal DNA was found in the infected grain, which, for *F. culmorum*, ranged from 8.87 ± 0.87 to 205.01 ± 8.81 ngDNA/μL. The level of grain infection by *F. graminearum* expressed by the amount of DNA determined by qPCR was lower and ranged from 1.58 ± 0.18 to 108.69 ± 14.59 ngDNA/μL. No infection was detected in the control grain.

The consequence of grain infection was contamination with DON and ZEA mycotoxins. In the case of *F. culomorum*, the levels ranged from 12.07 ± 0.16 μg/g to 19.33 ± 0.08 μg/g DON and from 2.10 ± 0.03 [μg/g] to 3.33 ± 0.18 μg/g ZEA in individual repetitions. Although the qPCR method showed that the grain infection with *F. graminearum* was lower than that of *F. culmorum*, HPLC analyses revealed higher grain contamination with DON, which ranged from 10.85 ± 0.13 to 28.05 ± 0.39 μg/g.

Contamination of UAE grain due to infection by *F. culmorum* was similar to *F. graminearum* and ranged from 1.99 ± 0.11 to 3.99 ± 0.11 μg/g. As a result of the analysis, no significant relationship was found between the amount of DON and ZEA determined in the grain (Figure 5).

### 3.2. GC-MS Analysis of Volatile Compounds

**Figure 6:**
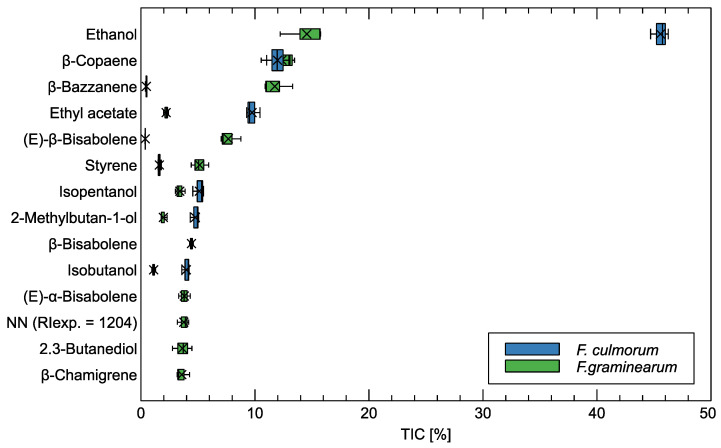
Most abundant volatile organic components recognized by the GC-MS method in samples of *Fusarium* species. The plotted quantity TIC [%] is the percentage of a given component in the total ion current of the measured sample. In the plots, boxes span from 1st to 3rd quantile, with the median indicated by a line and whiskers indicating 1.5 inter-quantile range.

In Figure 6, we present the main chemical components identified by the GC-MS analysis. We restrict the list of components to those for which the mean of their presence in at least one of the studied fungi species was above 3% of the total quantity of molecules detected in the sample. We determined the quantity of the chemical components present in the sample as the ion current collected during the measurement of the sample. Table A2 in Appendix C presents the full list of the chemical components identified.

The main component detected in the wheat infected by both of the studied *Fusarium* species is ethanol. Its presence in the *F. culmorum* samples had a much higher percentage than in the case of the *F. graminearum* samples. What should be understood, looking at the results, is that the presented percentage does not indicate the absolute magnitude of the ethanol concentration found in the samples. Roughly speaking, the percentage is calculated as the relation of the number of ethanol molecules (measured by the ion current of its chromatography peak) to the number of molecules of all chemical compounds detected in the sample (measured as the total ion current of the sample).

Sesquiterpenes were the main group of chemical compounds detected in the volatile composition of *F. greaminarum*, and their total content was 55.86 ± 1.72%. Sesquiterpenes, which occurred in the highest concentration, were *β*-copaene (12.36 ± 1.29%), *β*-bazzanene (11.74 ± 1.11%), and (E)-*β*-bisabolene (7.67 ± 0.77%). The content of sesquiterpenes in volatile emissions from *F. culmorum* was 16.78 ± 1.05%. Germacrene D, *α*-acoradiene were identified in both fungi, whereas *β*-bisabolene, (E)-*α*-bisabolene, *β*-chamigrene, and *α*-barbatene were found only in *F. greaminarum*.

Monoterpenes, such as myrcene, limonene, and *α*-terpinolene, occurred only in *F. greaminarum*, while *δ*-3-carene was identified in both species of *Fusarium* fungi.

Volatile compositions of *F. culmorum* and *F. greaminarum*, respectively, contained 62.52 ± 1.40% and 26.90 ± 0.90% alcohol. They were especially rich in ethanol, with concentrations of 45.56 ± 0.65% and 14.55 ± 1.65%, respectively. Alcohols such as propan-1-ol, isobutanol, isopentanol, and 2-methylbutan-1-ol were found in two *Fusarium* species; 2,3-Butanediol, oct-1-en-3-ol, benzeneethanol and nonan-1-ol occurred only in emission from *F. greaminarum*, whereas 2-ethyl-hexan-1-ol was in *F. culmorum*.

Benzeneacetaldehyde was identified in both analyzed species of *Fusarium*. The presence of aliphatic aldehydes, such as isopentanal and 2-methylbutanal, was a characteristic feature of the chemical composition of *F. culmorum*. Ketones including nonan-2-one, undecan-2-one, tridecan-2-one, and two isomers of 3-hydroxybutan-2-one were specific compounds for *F. greaminarum*. The content of esters in *F. culmorum* (13.96 ± 0.43%) was four times higher than that in *F. greaminarum* (3.21 ± 0.15%). Ethyl acetate, isobutyl acetate, isopentyl acetate, and 2-methylbutyl acetate were detected in both *Fusarium* fungi. Ethyl propanoate (0.34 ± 0.06%) and propyl acetate (0.27 ± 0.01%) occurred only in *F. culmorum*, while n-nonanyl acetate (0.19 ± 0.03%) occurred in *F. greaminarum*. Additionally, in the composition of volatiles emitted from *F. culmorum* and *F. greaminarum*, acetic acid and styrene were identified. The presence of pyridine (1.13 ± 0.05%) was found only in *F. culmorum*.

As one can notice, most of the components detected were present in the *F. graminearum* samples, which may indicate that this fungal species produced a highly varied set of volatile components.

### 3.3. Electronic Nose Measurements

The sensor response pattern presented in Figure 3 does not demonstrate that the sensor’s response allows differentiation between samples infected by *Fusarium* and healthy ones. The intention of presenting this figure was to illustrate an example of a response of a single sensor during one measurement cycle.

Sensor sensitivity to the presence of volatiles emitted by *Fusarium* fungi consists of the fact that the sensor response is different when we compare it to the response in the presence of volatiles emitted by healthy grains. We did not compare the sensors’ responses to various samples in Figure 3, as that would make it difficult to demonstrate the idea of the pattern collected during one measurement cycle. However, we can visualize and demonstrate the differences between responses in the presence of *Fusarium* and the presence of healthy grain by examining the features extracted from the raw sensors’ response curves.

More advanced and quantitative analysis of differentiation between samples consists of building machine learning models, the results of which we present in Section 3.3.3.

#### 3.3.1. Sensor Response during the Gas Adsorption Phase

**Figure 7:**
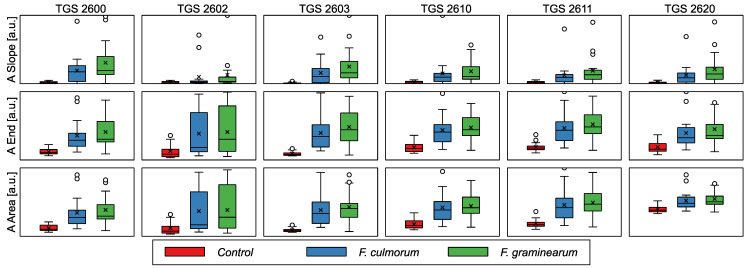
Comparison of features extracted from the adsorption phase of the sensor response for three studied categories of samples. In the plots, boxes span from 1st to 3rd quantile, with the median indicated by a line, whiskers indicating 1.5 inter-quantile range, the × symbol indicating the mean value, and empty circles beside whiskers indicating the outlier observations.

In Figure 7, we present a comparison of the sensor response for three studied categories of samples: a control group of healthy grains and two groups of samples containing wheat infected by two *Fusarium* pathogenic fungi species, *F. culmorum* and *F. graminearum*. A comparison of the distribution of responses is presented using a box-plot chart, which allows one to visualize some of the main characteristics of the data distribution.

Several patterns can be recognized in these data. Firstly, there is a visible difference between healthy and infected samples. In addition, we cannot detect a significant difference between samples infected with two types of *Fusarium*. These patterns suggest that it should be possible to recognize the infected state of the grains from the electronic nose data, but distinguishing between the *Fusarium* species may be difficult.

Another pattern observed in this visualization is a significant difference in the data scatter between healthy and infected samples. The measurements of healthy wheat show a relatively low variance, while the observations obtained for infected samples show much greater scatter. The nature of the infected samples can explain such an effect. The fungal growth on independent Petri dishes was different and different amounts of volatiles were produced, resulting in a different response from the sensors. Even if the measurements were carried out over several days, the same Petri dish could give different results, as the growth process during one day could lead to differences in the samples and their odor. However, apart from these differences, the overall patterns allow us to distinguish between healthy and infected samples. Furthermore, it seems that differentiation between the samples should be possible based on the data collected by an individual sensor.

#### 3.3.2. Sensor Response during Temperature Modulation Phase

**Figure 8:**
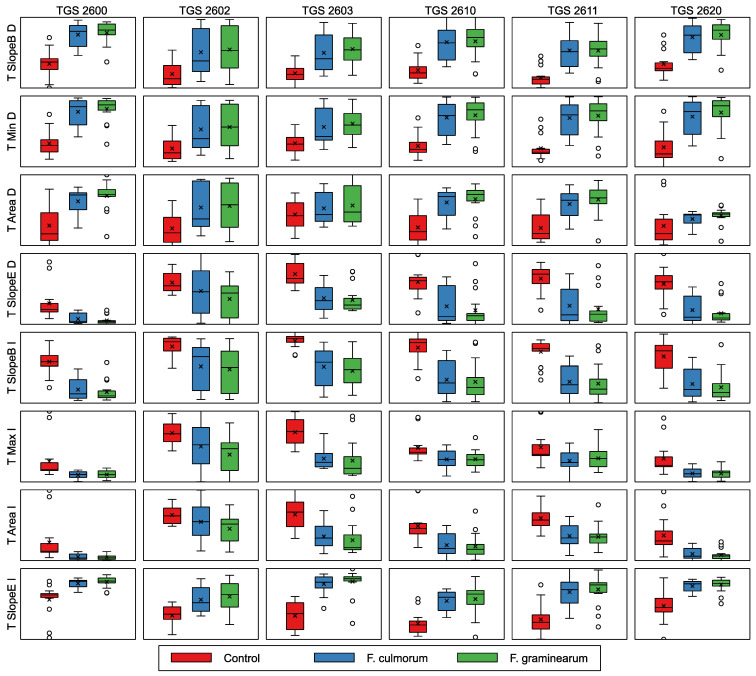
Comparison of features extracted from the sensor response during the sensor temperature modulation phase (duration of temperature modulation step of 100 time units) for three studied categories of samples. In the plots, boxes span from 1st to 3rd quantile, with the median indicated by a line, whiskers indicating 1.5 inter-quantile range, the × symbol indicating the mean value, and empty circles beside whiskers indicating the outlier observations.

In Figure 8, we present a distribution of features extracted from the sensor response curves during the first period of temperature modulation. As an example, we present the data for the case of a modulation step duration of 100 time units (75 s).

Similar patterns to those observed for the patterns extracted from the adsorption phase of the response can be noticed. There is a visible difference between the control group and infected samples, and no clear difference between the two categories of *Fusarium* species.

#### 3.3.3. Classification Models Using Electronic Nose Data

In the previous section, we presented distributions of the features extracted from the sensor response curves for three studied categories of samples. These results indicated that there is a noticeable difference between healthy grains and those infected by *Fusarium* fungi. However, examining the feature distribution gives only qualitative knowledge about the differences. We trained several classification models using the Random Forest machine learning algorithm to assess the differences quantitatively and measure the possibility of using electronic nose measurements to detect wheat grain infection.

**Figure 9:**
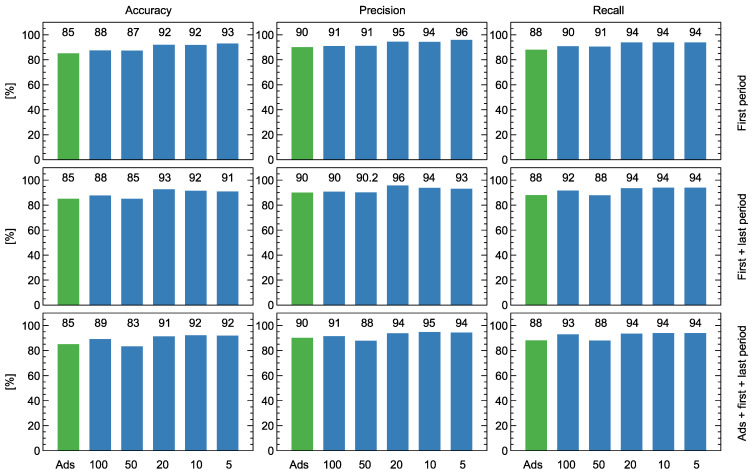
Classification models’ performance trained with various sets of predictors. The first row of subfigures presents the models using features extracted from the first repetition of the sensor heater voltage modulation. The second row of subfigures presents models when data from the first and last repetitions of the sensor heater voltage modulation were used to extract predictors. The last row of subfigures presents model performance, using as predictors data from the first and last repetition of the heater voltage modulation as well as features obtained from the adsorption phase of the sensor response. The green bars represent the performance of the model when, as predictors, only the features extracted from the adsorption phase of the sensor response are used. The length of the sensor heater voltage modulation step is indicated on the x-axis. Various measures of the performance are indicated above the columns of the subfigures.

In Figure 9, we compare the classification performance of several variants of models trained with various subsets of the predictors.

First of all, the classification’s performance is presented when we use features extracted from the adsorption phase of the sensor response as the predictors. The overall performance of this model gave an accuracy of 85%, precision of 90%, and recall of 88%. In our analysis, this will be treated as the baseline level to which we will compare the other results.

The other set of trained models was used to predict the features extracted from the sensor response curves collected during the modulation of the sensor heater voltage. We can observe in this figure that the overall performance of the models using such features as predictors exhibits better performance than those based on the data collected during the adsorption phase. We observed similar results in one of our previous experiments [31].

In our experiment, we used various lengths of the modulation step, and the modulation was repeated several times until the subsequent repetitions were very similar.

In the first row of subfigures in Figure 9, we present the performance of the models based on features extracted from the first period of the modulation. That means both from the step after the decrease and increase in the voltage, as presented schematically in Figure 4 and Table 1. As one can observe, we slightly improved the classification performance when the modulation step’s length was shorter, and the best classification results reached 92% accuracy, 94% precision, and recall for the length of modulation of 20 time units (15 s). A slightly better precision of 96% was obtained for the precision for the modulation step of 5 time units (3.75 s). However, in our opinion, recall is the most important measure of classification performance in the studied case as it estimates the possibility of detecting infection.

Another hypothesis we wanted to investigate was whether several repetitions of the sensor heater voltage modulation can bring more useful information, allowing us to differentiate between healthy and infected samples. As we presented in Figure 3 and Figure A1 in Appendix B, the first period of the repetition has a different shape than the subsequent. For that reason, we decided to use, as predictors for the classification model, the features extracted both from the first and the last repetition of the modulation. In the second row of subfigures in Figure 9, we present the performance of such models. As we can see, we have not improved the accuracy or recall of the classification performance. A slightly better precision could be obtained for the duration of the modulation step of 20 time units (15 s). However, as we already noticed, since recall is the primary measure of the model performance that should be considered in this type of experiment, we can conclude that there was no observed improvement in the classification performance when sensor heater voltage modulation was performed several times and features extracted from such data augmented the set of model predictors.

Another set of models we investigated used as predictors features extracted from the first and last repetition of the sensor temperature modulation and the features extracted from the adsorption phase of the sensor response curve. We decided to investigate such a combination of the modeling features, as the sensor response during these two phases is governed by different physical mechanisms (change in gas composition versus change in sensor operating temperature). That may suggest that some additional features allowing for improved classification performance could be discovered by the model using such a combined set of features.

In the third row of the subfigures in Figure 9, we compare the classification performance obtained by such a series of models. As one can see, such data fusion did not improve classification performance.

## 4. Discussion

### 4.1. Operation of MOX Gas Sensors in Temperature Modulation Mode

Most commercially available MOX gas sensors that can be used to build a low-cost electronic nose are designed to operate at a constant sensor heating voltage. This is the case with the Figaro Inc. sensors [32] used in our device. However, there are many research studies [24,25,31,38,39] that report on electronic noses based on the Figaro TGS series sensors using the possibility of gas detection in the sensor temperature modulation framework. An important aspect of operating the electronic nose in this mode is the protocol choice for the sensor heating modulation profile.

Commonly used approaches are different variants of the abrupt change in the heating voltage [38,40,41,42,43,44,45,46,47], which should allow the capture of the response of the sensor to such a change in operating conditions. In most of these studies, rectangular or staircase modulation profiles were used. Hosseini-Golgoo et al. [43,48] proposed a staircase profile in which the heating voltage of the sensor was increased from 1 to 5 V in several steps. A similar modulation profile was used by Liu et al. [47]. Amini et al. [46] and Gosangi et al. [45] investigated the response of the sensors to an increasing heating voltage by using the protocol of rectangular steps with different heights. Hossein-Babaei et al. [41,49] proposed a rectangular sensor heater voltage modulation profile with a constant high but varying base voltage level. This protocol was applied to a low-cost generic tin oxide-based gas sensor. Zhao et al. [50] used a rectangular profile of sensor temperature modulation applied to a SnO_2_ sensor for toxic and combustible gas detection. Duran et al. [38] used a rectangular shape of the sensor heating modulation profile throughout the transition range of the sensor response to the change in gas composition from clean air to the odor conditions studied.

Another group of approaches utilized sensor heater voltage profile variants with sinusoidal modulation [39,51,52,53,54]. In our opinion, this approach is more suited to cases where several repetitions of the modulations are performed, and the extraction of the characteristics of the response is based on the spectral analysis of the signals. Llobet et al. [51] investigated the selection of modulation frequencies for temperature-modulated gas sensors by noise methods. Vergara et al. [52] reported experimental results where the working temperature of MOX sensors was modulated with optimized multi-sinusoidal signals and showed that the features extracted during temperature modulation reduced data scatter in repeated measurements. Oates et al. [55] reported a low-cost electronic nose with a sinusoidal protocol of sensor heating voltage applied to commercially available sensors used to classify oil types and later demonstrated [56] its application to the detection of different types of food. Wozniak et al. [39] performed an FFT analysis of the temperature-modulated response of semiconductor gas sensors for the prediction of ammonia concentration under the influence of humidity and validated the performance of the classification after a delay of 100 days. Herrero-Carron et al. [54] proposed an approach to develop a temperature-modulated strategy that adapts to the online activity of the sensor.

Other patterns of modulating the temperature of the sensor heating voltage have also been reported. Yin et al. [57] proposed a triangular shape of sawtooth-like modulation applied to TGS 2602 and TGS 2620 series sensors. Krivetskiy et al. [58] reported on a triangular modulation profile. Yuan et al. [59] proposed a triangular in combination with rectangular profiles, starting from a base level with low sensor temperature conditions. He et al. [60] used a spike-like modulation protocol with a variable frequency of spikes during the measurement process. Iwata et al. [61] proposed the use of a protocol with a modulation profile for the sensor heater voltage in which amplitude and frequency changed periodically.

In our previous research [24,25,31], we used a rectangular form of sensor heating voltage modulation that uses a single repetition of the voltage change step [24]. We have verified that voltage modulation with a relatively small voltage drop from the nominal sensor heater is the correct choice and allows us to perform better in classifying samples using electronic nose data.

In our previous research, as well as in the research reports of other authors [40,41,42,43,44,45,46,47], in most cases, the length of the heating voltage step (corresponding to the frequency of the heating voltage modulation) was chosen so that the sensor reached a steady state at the end of its duration, and only then were the further modulation steps performed. The choice of such a length of the modulation step is logical, as it makes it possible to obtain response results that are determined by the factors of the presence of the gas and the level of the heating voltage and are not strongly influenced by the sensor response during the previous modulation step. As can be seen in Figure 3 and in more detail in Figure A1 in Appendix B, the subsequent periods of modulation repetitions differ if we use a shorter length of the heater voltage modulation step and start the subsequent modulation before the sensor response reaches a steady state. This effect is more pronounced the shorter the modulation steps are (i.e., the higher the modulation frequency is), as the subsequent modulation periods accumulate part of the response from the previous periods.

Some studies have shown that the electronic nose sensor captures the most meaningful information that can be used to differentiate between sample categories right at the beginning of the transition phase [31,62]. This is a very different approach from many other research studies and applications of electronic noses, where the steady state after the completion of the transient phase of the response is used for classification.

In the present manuscript, we followed the paradigm of applying the fast detection approach and, therefore, verified that shortening the modulation frequency period does not affect the performance of the classification models. We also found that adding more repetition periods did not improve the classification performance.

### 4.2. Detection of *Fusarium* Pathogenic Fungi Using Electronic Nose Measurements

*Fusarium circinatum*, a fungal pathogen that is lethal to many pine species (*Pinus*), could cause significant economic and ecological losses, especially if it were to spread in Europe. The above-mentioned devices based on the emission of volatile organic compounds (VOCs) can be used for the early detection of pathogens and accelerate the implementation of measures to mitigate the consequences of the unwanted introduction of new organisms [63].

Gas chromatography with VOC mass spectrometry enables automated data analysis and machine learning, which in turn can distinguish diseased from healthy seedlings. In the case of *P. pinea* and *P. radiata*, this differentiation was possible even in non-symptomatic seedlings with latent infection in healthy-looking plants. In vitro analyses showed that even closely related *Fusarium* species can be distinguished based on their VOC profiles, further supporting the method’s potential for early disease detection and diagnosis [63].

Maize crops are easily spoiled by fungi with potential mycotoxin contamination. One of the most common toxins is fumonisin B1, mainly produced by *Fusarium verticillioides* and associated with human and animal diseases. The volatile fungal compound formula (headspace) can be used as a marker for toxin production. In the present work, we have shown that the electronic nose can detect fungal contamination of maize and classify *F. verticillioides* strains concerning their different fumonisin production behavior. The electronic nose could be an important method to analyze maize to prevent mycotoxins from entering the food chain [64].

The results obtained in our experiment can be compared with other results reported for detecting *Fusarium* fungal infections in different cereals or other foods.

Gancarz et al. [20,21] investigated the possibility of using an electronic nose to monitor the development of the fungal microflora of rapeseed (during the first eighteen days of storage). A Cyranose 320 instrument from Sensigent was used to analyze the volatile organic compounds. Each sample of infested material was divided into three sections, and the degree of spoilage was measured in three ways: the analysis of colony-forming units, the determination of ergosterol content, and the measurement of volatile organic compounds using the e-nose. The generated signal patterns were subjected to principal component analysis, and six groups with different degrees of spoilage were identified. Sensogram analysis was performed for multiple sensors with a strong signal for each oilseed rape spoilage group. The ratio of association time to steady state was calculated. This ratio was different for low and highest ergosterol content and colony-forming units. The results showed that the e-nose can be a useful tool for the rapid assessment of oilseed rape spoilage.

Presicce et al. [12] reported qualitative evidence that it is possible to detect wheat grain infected by *F. poae* using their prototypical electronic nose. Similar research was carried out by Falasconi et al. [64] for the case of maize infected with *F. verticillioides*. Eifler et al. [8] reported the results of GC-MS and electronic nose detection of four species of *Fusarium* fungi (*F. graminearum, F. culmorum, F. cerealis*, and *F. redolens*) infecting wheat grain. Their electronic nose was able to distinguish between four species with an accuracy of more than 80%.

What is interesting to note is that the authors in Ref. [8] (Figure 1A) have a scatter plot with latent variables showing the distribution of the analyzed samples. It can be observed that the species of *F. graminearum* and *F. culmorum* detected in the present study are very close to each other, which also indicates that it may be difficult to distinguish between the species using electronic nose data. The list of chemical components reported by these authors that were detected in the samples of these two species is also very similar.

Feng et al. [65] reported early detection of *Fusarium oxysporum* infection in tomato processing using a low-cost portable electronic nose with a classification accuracy in the range of 84–95%.

Labanska et al. [66] reported the detection of *F. oxysporum* basal rot infections in onions and shallots using the PEN3 electronic nose with classification accuracy of 87–97%.

Camardo Leggieri et al. [67] reported exploring the e-nose in conjunction with CART, a machine learning approach to detect *F. graminearum* in wheat. They used the commercially available electronic nose device PEN3 (Airsense Analytics GmbH, Schwerin, Germany) and achieved a detection accuracy of 88–92%. This encouraging result is comparable to our results obtained with an inexpensive, home-made device.

## 5. Summary and Conclusions

Wheat grain samples were prepared by infecting them with *Fusarium culmorum* and *F. graminearum* pathogenic fungi. Samples with visible symptoms were used for further investigation using various methods.

The presence of pathogens was confirmed with genetic qPCR tests, and the amount of DNA in samples was measured. The concentration of deoxynivalenol (DON) and zearalenone (ZEA) toxins in the samples was measured using HPLC analysis. In all infected samples, toxin presence was detected with a similar order of magnitude in the range of 10–30 μg/g for DON and 2–4 μg/g for ZEA.

Headspace solid-phase microextraction coupled with gas chromatography and mass spectrometry (HS-SPME/GC-MS) analysis of volatile organic compounds emitted by the two studied species of *Fusarium* fungi was performed. For both fungi, ethanol was the most abundant component that was produced, followed by *β*-kopaene. It has been found that *F. graminearum* produces a much more varied composition of chemical components.

A custom-made, low-cost electronic nose was used to collect data that could be used to differentiate between healthy and infected samples. The electronic nose device used applied a set of six commercially available MOX sensors from the Figaro Inc. TGS series. Two general methods of generation of transient sensor response were applied: (i) response to change in measured gas composition from clean air conditions to the studied odor, and (ii) response to modulation of sensor temperature by changes in the sensor heater voltage. The rectangular modulation protocol was used with the upper level at the nominal voltage of 5 V, as recommended by the manufacturer, and a voltage drop to a 4.5 V level. Various lengths of the modulation steps were used with several repetitions until the sensor response exhibited a stable pattern.

The Random Forests machine learning method was applied to build a classification model using the predictor set of features describing the shapes of the sensors’ responses. The detection of the infected samples with the recall at a level of 94% was achieved. The obtained classification performance is comparable to the results reported by other authors for studies on the detection of *Fusarium* infection, which were usually obtained using sophisticated commercial electronic nose devices.

It has been demonstrated that using sensor temperature modulation brings better results, allowing classification performance to be obtained. An improvement in the classification results was observed with a shorter length of the sensor heater voltage modulation step. It has been verified that the first period of the sensor heater voltage modulation allows for the collection of the best data for classification and that merging it with the data obtained from later repetitions did not improve it further. It has also been observed that the fusion of data from the sensor adsorption response range (response to change in gas composition) and sensor temperature modulation does not improve the classification.

These observations allowed us to conclude that the optimal choice of the mode of operation of the electronic nose device used is sensor heater temperature modulation with a short modulation step length.

## Figures and Tables

**Table 1:** Description of features extracted from the sensor response curve (Figure 4).

	Adsorption Phase
A_Slope	Slope of the response curve at the beginning of the measurement, just after opening the sensor chamber and exposing the sensors to the measured gas.
A_End	Sensor response at the end of the adsorption phase.
A_Area	Area under the curve for the adsorption phase of the measurement. It is equivalent to the average of the responses during that measurement phase.
	**Heater Voltage Drop Step**
T_SlopeB_D	Slope of the sensor response curve at the beginning of the temperature modulation phase.
T_Min_D	Minimum magnitude of the sensor response curve.
T_Area_D	Area under the sensor response curve.
T_SlopeE_D	Slope of the sensor response curve at the end of the heater voltage drop step.
	**Heater Voltage Increase Step**
T_SlopeB_I	Slope of the sensor response curve at the beginning of the heater voltage increase step.
T_Max_I	Maximum magnitude of the sensor response curve.
T_Area_I	Area under the sensor response curve.
T_SlopeE_I	Slope of the sensor response curve at the heater voltage increase step.

## Data Availability

Data are contained within the article.

## Appendix A. Electronic Nose Sensors

**Table A1:** List of sensors used in the electronic nose and target gases and odors [32].

Sensor	Target Detection
TGS 2600	Highly sensitive to low concentrations of gaseous air contaminants such as hydrogen and carbon monoxide, for example, in cigarette smoke. Can detect hydrogen at a level of several ppm.
TGS 2602	Highly sensitive to low concentrations of odorous gases such as ammonia and H_2_S generated from waste materials in office and home environments. Highly sensitive to low concentrations of VOCs such as toluene emitted from wood finishing and construction products.
TGS 2603	Highly sensitive to low concentrations of odorous gases such as amine-series and sulfurous odors generated from waste materials or spoiled foods such as fish.
TGS 2610	Uses filter material, eliminating the influence of interference gases such as alcohol, which is highly selective to LP gas.
TGS 2611	Uses filter material, eliminating the influence of interference gases such as alcohol, which is highly selective to methane gas.
TGS 2620	Has high sensitivity to organic solvents and other volatile vapors, making it suitable for organic vapor detectors/alarms.

## Appendix B. Sensor Response to Heater Temperature Modulation

In Figure 3, we presented the whole response of a sensor during one cycle of measurements. However, the scale of that figure does not allow us to see all the interesting details of the collected data. Thus, in Figure A1, we present fragments of the response during all sensor heater temperature modulation phases with various frequencies. We also present the shape of the collected response during the first repetition of each cycle.

## Appendix C. Results of the GC-MS Measurements

**Table A2:** List of chemical compounds identified by the gas chromatography–mass spectrometry measurements. The average value of the percentage of total ion current (TIC) is presented together with standard deviation obtained from measurements of three independent samples.

Compounds and Groups of Compounds	Content (% of TIC)	
	***F. culmorum***	***F. greaminarum***
**Sesquiterpenes**	**16.78 ± 1.05**	**55.86 ± 1.72**
*α*-Funebrene	-	0.37 ± 0.05
*α*-Barbatene	-	2.15 ± 0.12
Sesquiterpene (RIexp. = 1419)	1.04 ± 0.23	1.93 ± 0.17
*β*-Copaene	11.98 ± 0.80	12.36 ± 1.29
Isobazzanene	-	0.43 ± 0.04
(E)-*β*-Famesene	-	1.73 ± 0.07
*β*-Santalene	-	0.29 ± 0.04
*α*-Acoradiene	0.53 ± 0.05	0.47 ± 0.00
*β*-Acoradiene	-	1.07 ± 0.14
*β*-Chamigrene	-	3.57 ± 0.49
Sesquiterpene (RIexp. = 1484)	-	1.00 ± 0.10
Germacrene D	1.45 ± 0.20	0.88 ± 0.10
*α*-Cuprenene	-	1.42 ± 0.02
*α*-Chamigrene	-	0.54 ± 0.07
Sesquiterpene (RIexp. = 1513)	0.92 ± 0.12	-
*β*-Bisabolene	-	4.43 ± 0.14
*β*-Bazzanene	0.47 ± 0.06	11.74 ± 1.11
(E)-*β*-Bisabolene	0.37 ± 0.01	7.67 ± 0.77
(E)-*α*-Bisabolene	-	3.81 ± 0.41
**Monoterpenes**	**0.57 ± 0.06**	**1.03 ± 0.10**
Myrcene	-	0.29 ± 0.03
*δ*-3-Carene	0.57 ± 0.06	0.20 ± 0.03
Limonene	-	0.23 ± 0.03
*α*-Terpinolene	-	0.31 ± 0.02
**Alcohols**	**62.52 ± 1.40**	**26.90 ± 0.90**
Ethanol	45.56 ± 0.65	14.55 ± 1.65
Propan-1-ol	2.34 ± 0.23	1.07 ± 0.07
Isobutanol	3.98 ± 0.29	1.11 ± 0.13
Isopentanol	5.12 ± 0.41	3.40 ± 0.35
2-Methylbutan-1-ol	4.78 ± 0.32	1.96 ± 0.24
2.3-Butanediol	-	3.65 ± 0.70
Oct-1-en-3-ol	-	0.32 ± 0.08
2-Ethyl-hexan-1-ol	0.74 ± 0.08	-
Benzeneethanol	-	0.09 ± 0.02
Nonan-1-ol	-	0.75 ± 0.04
**Aldehydes**	**1.78 ± 0.15**	**0.25 ± 0.05**
Isopentanal	0.52 ± 0.04	-
2-Methylbutanal	0.49 ± 0.04	-
Benzeneacetaldehyde	0.77 ± 0.08	0.25 ± 0.05
**Ketones**	-	**2.19 ± 0.24**
3-Hydroxybutan-2-one, isomer 1	-	1.19 ± 0.27
3-Hydroxybutan-2-one, isomer 2	-	0.35 ± 0.05
Nonan-2-one	-	0.20 ± 0.03
Undecan-2-one	-	0.15 ± 0.03
Tridecan-2-one	-	0.30 ± 0.04
**Esters**	**13.96 ± 0.43**	**3.21 ± 0.15**
Ethyl acetate	9.74 ± 0.50	2.21 ± 0.18
Ethyl propanoate	0.34 ± 0.06	-
Propyl acetate	0.27 ± 0.01	-
Isobutyl acetate	0.48 ± 0.02	0.12 ± 0.01
Isopentyl acetate	1.37 ± 0.07	0.46 ± 0.05
2-Methylbutyl acetate	1.75 ± 0.05	0.23 ± 0.02
n-Nonanyl acetate	-	0.19 ± 0.03
**Other compunds**	**4.39 ± 0.24**	**6.46 ± 0.69**
Acetic acid	1.65 ± 0.32	1.32 ± 0.24
Pyridine	1.13 ± 0.05	-
Styrene	1.61 ± 0.12	5.14 ± 0.63
**Unidentified compunds**	**-**	**4.10 ± 0.41**
NN (RIexp. = 1175)	-	0.35 ± 0.02
NN (RIexp. = 1204)	-	3.76 ± 0.42
